# Exhaled volatile substances mirror clinical conditions in pediatric chronic kidney disease

**DOI:** 10.1371/journal.pone.0178745

**Published:** 2017-06-01

**Authors:** Juliane Obermeier, Phillip Trefz, Josephine Happ, Jochen K. Schubert, Hagen Staude, Dagmar-Christiane Fischer, Wolfram Miekisch

**Affiliations:** 1 Department of Anesthesiology and Intensive Care Medicine, Rostock Medical Breath Research Analytics and Technologies (ROMBAT), University Medicine Rostock, Rostock, Germany; 2 Department of Pediatrics, University Medicine Rostock, Rostock, Germany; Helsingin Yliopisto, FINLAND

## Abstract

Monitoring metabolic adaptation to chronic kidney disease (CKD) early in the time course of the disease is challenging. As a non-invasive technique, analysis of exhaled breath profiles is especially attractive in children. Up to now, no reports on breath profiles in this patient cohort are available. 116 pediatric subjects suffering from mild-to-moderate CKD (n = 48) or having a functional renal transplant KTx (n = 8) and healthy controls (n = 60) matched for age and sex were investigated. Non-invasive quantitative analysis of exhaled breath profiles by means of a highly sensitive online mass spectrometric technique (PTR-ToF) was used. CKD stage, the underlying renal disease (HUS; glomerular diseases; abnormalities of kidney and urinary tract or polycystic kidney disease) and the presence of a functional renal transplant were considered as classifiers. Exhaled volatile organic compound (VOC) patterns differed between CKD/ KTx patients and healthy children. Amounts of ammonia, ethanol, isoprene, pentanal and heptanal were higher in patients compared to healthy controls (556, 146, 70.5, 9.3, and 5.4 ppbV vs. 284, 82.4, 49.6, 5.30, and 2.78 ppbV). Methylamine concentrations were lower in the patient group (6.5 vs 10.1 ppbV). These concentration differences were most pronounced in HUS and kidney transplanted patients. When patients were grouped with respect to degree of renal failure these differences could still be detected. Ammonia accumulated already in CKD stage 1, whereas alterations of isoprene (linked to cholesterol metabolism), pentanal and heptanal (linked to oxidative stress) concentrations were detectable in the breath of patients with CKD stage 2 to 4. Only weak associations between serum creatinine and exhaled VOCs were noted. Non-invasive breath testing may help to understand basic mechanisms and metabolic adaptation accompanying progression of CKD. Our results support the current notion that metabolic adaptation occurs early during the time course of CKD.

## Introduction

In patients with end-stage chronic kidney disease (CKD) a "uremic fetor" is frequently noted and has been assigned mainly to exhalation of ammonia, dimethylamine and trimethylamine [1]. Since then, several studies on the breath profile of volatile organic compounds (VOCs) have been performed with adults on hemodialysis [2–7] and evidence was obtained that the breath profile is already affected as renal function is only mildly impaired [5].

As kidney dysfunctions lead to an impaired removal of waste products from the blood, urea and creatinine among others are metabolized to ammonia. Excess ammonia as well as nitrogen-containing volatile compounds like methylamines can diffuse into the lungs and are shown in higher concentrations in breath of end-stage CKD patients in comparison to healthy controls [4,8].

Increased concentrations of isoprene in breath have been described in patients with terminal renal failure. Exhaled isoprene concentrations were higher after hemodialysis [2,3,8].

Aldehydes such as malondialdehyde, pentanal and hexanal have been proposed as markers for oxidative stress, which is correlated to kidney diseases [9,10]. Dimethyl sulfide, methyl propyl sulfide, allyl methyl sulfide, thiophene and benzene changed their blood and breath levels during the hemodialysis treatment, whereas cyclohexanone and 2-propenal have been described as uremic toxins [7].

Whereas in the vast majority of adult CKD patients ageing and/or life-style associated comorbidities have to be considered and may bias the results, these issues are less relevant in pediatric CKD patients. Breath gas analysis appears as an attractive diagnostic tool as it is completely non-invasive. Up to now, different analytical techniques were applied to detect changes in breath profiles in this patient group. These techniques comprise hyphenated techniques such as GC-MS or direct MS able to identify and quantify large numbers of potential marker compounds, some others such as sensor technologies are better suited for point of care use and are still in early stages of development [6,11]. Despite these developments, data on exhaled volatile organic compounds in children are limited and refer to children with either inflammatory bowel diseases, chronic liver disease or respiratory diseases, while data on children with chronic kidney diseases are not available thus far [12–14].

We applied proton-transfer-reaction time-of-flight mass spectrometry (PTR-ToF-MS) for real time analysis of VOCs in exhaled breath of pediatric patients with mild-to-moderate CKD and healthy controls matched for age and sex. This technique gives rise to the detection of a broad panel of volatile organic compounds ("volatilome"). The present study was directed to characterize breath profiles from healthy children and pediatric patients with mild-to-moderate CKD.

## Methods

### Patients and controls

The study received appropriate approval from the institutional review board (Rostock University Medical Centre Ethics committee) and was performed in accordance with the Declaration of Helsinki. Subjects and/or their parents gave assent written informed consent prior to participation.

All children aged 4 to 18 years, suffering from CKD stage 1–4 on conservative treatment, or after kidney transplantation (KTx) and being treated at our institution were eligible for this study. For the classification of CKD stages, the definition from Kidney Disease: Improving Global Outcomes (KDIGO) of 2012 was used. Children with acute infections as well as those with upper airway infections, metabolic disorders, chronic inflammatory or hepatic disease were excluded. A total of 56 patients (36 boys) consented to participate and was enrolled during an 18 months study period. The Schwartz formula was employed to estimate individual glomerular filtration rates (eGFR) [15]. Serum creatinine was determined according to Jaffé [16]. Demographic and clinical data including the history of disease were gathered by interview and chart review, respectively. Age- and sex-matched healthy controls (n = 60; 28 boys) were recruited from local schools and a trained physician measured weight and height throughout the study using electronic scales and a fixed stadiometer. The LMS method was used to calculate standard deviation scores (SDS) of the body mass index (BMI-SDS) according to Kromeyer-Hauschild et al. [17].

### Breath sampling

Patients’ breath sampling was consecutive to a routine visit in our outpatient clinic. Breath gas analysis was performed at room temperature (air-conditioned room at 22°C) and in a quiet environment, i.e. in the absence of powerful audio-visual and other mental stimuli, and all subjects were tested individually. Patients adhered to their individual therapeutic scheme.

**Fig 1:**
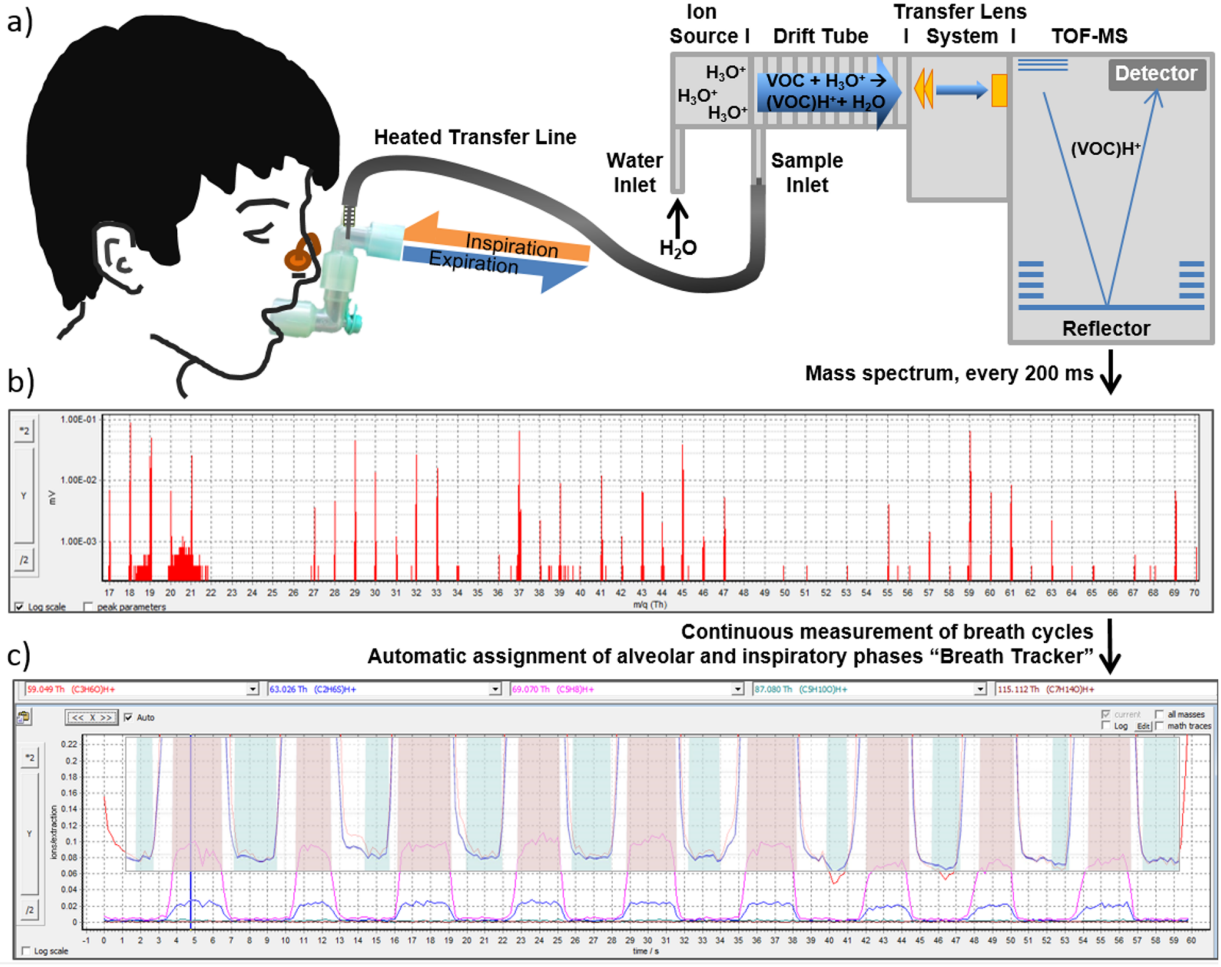
Schematic description of continuous real-time breath analysis. a) Participants breathed through a sterile mouthpiece without resistance. Ex- and inhaled breath was transferred continuously into the heated transfer line (connected via t-piece) of the PTR-ToF-MS in a side-stream mode at a flow of 20 ml/min. b) Every 200 ms a TOF—mass spectrum was recorded. c) Profiles of breath VOCs could be recorded continuously and in a phase resolved way. Acetone (red curve 59.049 m/z—as endogenous, blood borne VOC) was used to track breath phases and to assign all other mass traces to alveolar (red areas) and inhalation (blue areas) phases. In this way, intensities of VOCs other than acetone, such as isoprene (pink curve, 69.070 m/z) or dimethyl sulfide (blue curve, 63.026 m/z) could be assigned to the different breath phases and quantified in alveolar and inspiratory air.

Participants were asked to breathe evenly through the mouth into a sterile, exchangeable T-piece, which was connected via Luer lock adapter to the inert silcosteel transfer line (ID 0.75 mm, Restek, Bellafonte, USA) of the PTR-ToF-MS (Fig 1). A small part of ex- and inhaled breath (20 ml/min) was transferred to the PTR in a continuous side-stream mode. The transfer line temperature was 75°C to avoid condensation. The T-piece was upheld by participants to adjust a comfortable height.

Breathing through the mouth piece was without additional resistance. In some cases, children were holding their nose or used a nose clip during the measurement when having problems with breathing only through the mouth. Children were asked to breathe 5 min in a spontaneous and continuous way. Breath gas as well as room air were measured continuously for 5 min by means of PTR-ToF-MS. Generally, we analyzed data from the third minute, when most children had adapted to the procedure. Exhaled VOC concentrations were averaged over one minute and were then further analyzed.

### Measurement technique

Breath gas analyses were done by means of a Proton Transfer Reaction -Time of Flight—mass spectrometer (PTR-ToF-MS) 8000 (Ionicon Analytik GmbH, Innsbruck, Austria). Using soft ionization (proton transfer) PTR-ToF-MS allowed the direct and continuous detection of volatile compounds down to pptV concentrations at a high mass (~4000 m/dm) and time (~200 ms) resolution. Details of the PTR-ToF-MS technique applied were described before [18].

Briefly, within the drift tube of PTR-ToF-MS, the molecules (M) are ionized by transfer of H^+^ ions, which are produced from pure water vapor in the ion source (M + H_3_O^+^ → MH^+^ + H_2_O) (see Fig 1). The positively charged molecule ions are focused by a lens system and transferred to the ToF-MS. The mass analyzer is a high mass resolution, orthogonal acceleration, reflectron ToF-MS (Tofwerk AG). Here the ions are accelerated by a positive impulse and diverted by a magnetic field (Reflectron) and subsequently detected on a multichannel plate. Resulting flight times are recorded.

The drift tube voltage was 610 V at a pressure of 2.3 mbar resulting in an E/N ratio of ~138 Td (E = electric field strength, N = gas number density; 1 Td = 10^−17^ V cm^2^). We applied a time resolution of 200 ms for data acquisition and recorded the data by the associated ToF-DAQ Software. Every minute the mass scale was recalibrated. All VOCs were measured in counts per seconds (cps) and their intensities were normalized onto primary ion (H_3_O^+^) counts.

### Calibration procedure

PTR-MS is regarded as pseudo-absolute method, hence analyte concentrations can be calculated directly from the reaction rate constants k without prior calibration [19]. However, exhaled breath is water saturated and this may bias quantification. Therefore, a liquid calibration unit (LCU, Ionicon Analytic GmbH, Innsbruck, Austria) was used for external calibration of relevant volatile compounds. The total gas flow in the LCU was 1000 ml min^−1^, water flow was 50 μl min^−1^ (corresponds to ~47 g m^−3^ humidity) and the temperature was set to 75°C to guarantee a complete evaporation of the standard mixture and the water content. A humidity of ~47 g m^−3^ corresponds to approximately 100% relative humidity at 37°C (breath temperature) reflecting water saturation of the exhalation. Analyte concentrations in the physiological range (typically 1–100 ppbV) were prepared by varying nitrogen and analyte gas flow in a way that the total gas flow was kept constant.

Standards of isoprene, C_1_-C_10_ aldehydes, acetone, ethanol, dimethyl sulfide were prepared by means of the LCU and directly transferred to the PTR-ToF-MS in order to receive the analyte concentrations. Ammonia and methylamine concentrations were calculated directly from k rates [20].

### Data processing

A Matlab-based data processing algorithm (“breath tracker”, Matlab version 7.12.0.635, R2011a) was applied for automatically recognizing alveolar and inspiratory phases [18]. Acetone as an endogenous compound was used as tracker substance for the recognition of alveolar phases. The tolerance interval for alveolar phases was set to 10%. Thus, all intensities within a breathing cycle, which were higher than 90% of the maximum intensity of acetone, contributed to alveolar phases. For inspired phases, the tolerance interval was set to 2%. Thus, all intensities within a breathing cycle, which were lower than 2% of the maximum of acetone within that cycle were attributed to inspired air. A minimally required number of data points was defined as well. Expirations with less than two alveolar data points within one cycle were regarded as non-reliable. The determined phase resolution was applied to all m/q of interest.

After separating alveolar and inspiratory phases by means of the “breath tracker”, the ratio of inspiratory to expiratory counts was calculated in order to discriminate exogenous and endogenous VOCs. Based on this procedure 71 out of 360 masses were selected for further data analysis. Principal component analysis (PCA) in combination with full cross-validation was used to investigate relationships between individual VOC patterns. The VOC patterns from healthy controls and CKD patients were tested for differences related to anthropometric and clinical characteristic.

### Statistics

For statistical testing and visualization of results SPSS statistical package 22 (SPSS Inc. Chicago, Illinois, USA), Sigma Stat statistical package Version 3.5 and Sigma Plot Version 10 (Jandel Scientific Inc.) were used. Correlations between variables were assessed by bivariate linear regression analysis (Spearman Rank Order coefficient). Kruskal-Wallis analysis of variance on ranks followed by Dunn’s test for nonparametric values were performed to evaluate differences between groups. All p-values are two sided and a p-value below 0.05 was considered significant. Data are given as median and range unless mentioned otherwise.

## Results

### Characteristics of patients and controls

All participants completed the study examinations and although patients were slightly younger than healthy controls, the standardized anthropometric data are fairly comparable (Table 1). Detailed information on medication of different patients groups are shown in S1 Table. We used an eGFR of at least 90 ml/min/1.73m^2^ (CKD stage 1) as threshold to discriminate between mild and moderate CKD and summarized patients with a functional renal transplant as a separate group. Patients were treated at our institution for *i)* hereditary malformations (MF), i.e. congenital abnormalities of kidney and/or urinary tract (CAKUT; n = 22) or polycystic kidney diseases; *ii)* a history of haemolytic uremic syndrome (HUS; either shiga toxin induced (n = 7) or atypical) or *iii)* chronic glomerulonephritis/nephrotic syndrome with complete or partial remission (summarized as glomerular disease; GD) (Table 2). In two patients, CAKUT became clinically relevant at an age of 10 and 17 years with concomitantly short duration of disease prior to enrolment.

**Table 1:** Anthropometric data of the study population and relevant medications in CKD patients.

	Patients (36m / 20f)	Controls (28m / 32f)
Age [y]	12.0 (4.00–18.0)	13.5 (7.00–18.0)
absolute BMI [kg/m^2^]	18.6 (9.25–35.9)	19.8 (15.0–28.1)
standardized BMI [SDS]	0.12 (-7.47–3.14)	0.19 (-1.32–1.92)
eGFR [ml/min/1.73m^2^]	117 (22.8–200)	
Duration of disease [y]	6.92 (0.17–16.7)	
**Antihypertensive Drugs (yes / no)**	36/20	
ACE-Inhibitors	22	
AT1 receptor antagonists	14	
Beta blockers	13	
Alpha blockers	3	
Vasodilators	10	
Diuretics	15	
**Immunosuppressive therapy (yes / no)**	20/36	
Corticosteroids	11	
Calcineurin-Inhibitors	14	
Everolimus	1	
Mycophenolate mofetil	14	
Statins	5	
Erythropoietin	7	
Growth Hormone	1	
25-Hydroxyvitamin D / Calcitriol	25	

**Table 2:** Clinical characteristics of patients. Data is given as median and range. Superscripts denote significant differences between identically labelled groups.

	Therapy	
	Conservative (n = 48)	KTx (n = 8)
**All patients**		
Duration of disease / KTx [y]	5.75 (0.17–16.7)	6.29 (1.33–12.0)
Urea [mmol/l]	5.45 (1.79–25.8)	10.1 (6.75–23.1)
eGFR [ml/min/1.73m^2^]	126 (29–199)	63 (23–114)
CKD stage 1 / stage 2 / stage 3 / stage 4	38 / 6 / 3 / 1	
**Haemolytic uremic syndrome (n = 9)**		
Duration of disease [y]	1.75 (0.17–14.3) ^b)^	
Urea [mmol/l]	5.32 (3.44–11.1)	
eGFR [ml/min/1.73m^2^]	128 (40.3–186)	
CKD stage 1 / stage 2 / stage 3	7 / 1 / 1	
**Glomerular Disease (n = 15)**		
Duration of disease [y]	3.91 (0.83–13.3) ^a)^	
Urea [mmol/l]	5.73 (2.64–9.37)	
eGFR [ml/min/1.73m^2^]	125 (63–199)	
CKD stage 1 / stage 2	12 / 3	
**Malformation of kidney and/or urinary tract (n = 24)**		
Duration of disease [y]	8.9 (0.6–16.7) ^a^^,^ ^b)^	
Urea [mmol/l]	4.89 (1.79–25.8)	
eGFR [ml/min/1.73m^2^]	122 (29–184)	
CKD stage 1 / stage 2 / stage 3 / stage 4	19 / 2 / 2 / 1	

^a)^ p < 0.005;

^b)^ p < 0.01

### VOC analysis and breath profiles

#### Heat map and PCA

More than 300 VOCs were detected in the breath gas of pediatric CKD patients and healthy controls. After correction for potential contaminations (inspiratory > expiratory concentrations) and for limits of quantitation, 71 substances were analyzed by means of heat maps, PCA and subsequent statistical testing to evaluate whether VOC patterns differ between pediatric CKD patients and controls.

**Fig 2:**
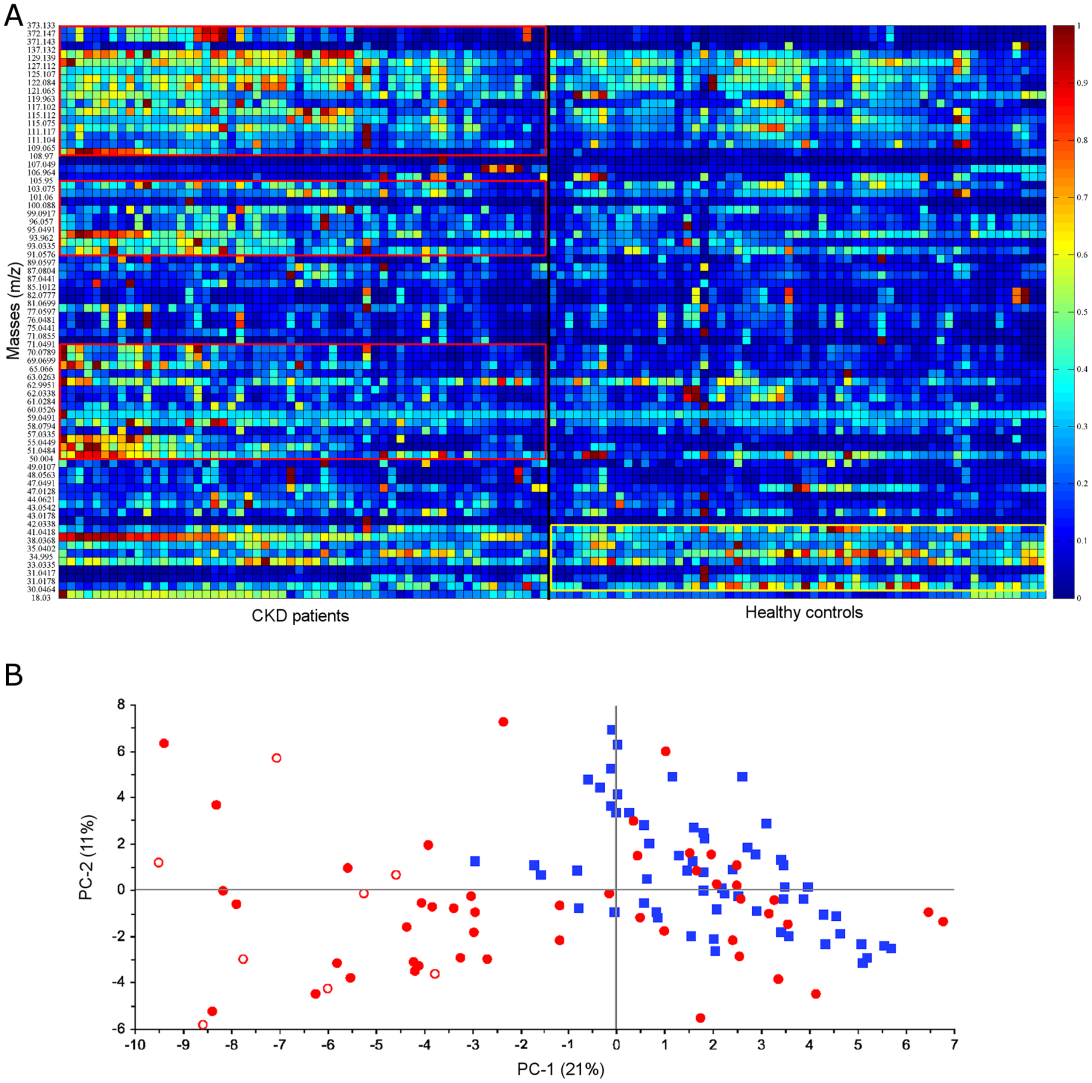
Heat map (A) and PCA score (B) obtained in CKD patients (n = 56) and controls (n = 60). A: Heat map based on normalized data of 71 mass traces (18 to 373 m/z) in breath of CKD patients (left) and healthy controls (right). Regions with elevated breath VOC levels are shown as red and yellow boxes for patients and controls, respectively. B: PCA score plot (PC-1 vs. PC-2) of healthy controls (blue squares, n = 60) and CKD patients (red dots, n = 56). Red circles represent data from patients with a functional renal transplant.

The normalized exhaled concentrations of 71 masses ranging from 18 to 373 m/z per participant are visualized as heat map (Fig 2A). At first glance, pediatric CKD patients exhaled higher amounts of VOCS with m/z above 50, whereas the relative concentration of VOCs with low mass is higher in healthy children.

A PCA including all participants and the selected 71 masses was calculated (Fig 2B). Healthy controls (blue squares) were compared with CKD patients (red dots). Healthy controls were mainly positively correlated on PC-1, whereas KTx patients were mainly negatively correlated on PC-1.

#### VOCs in CKD patients

On the basis of the PCA loading plot (S1 Fig), a Kruskal-Wallis analysis of variance on ranks was performed with exhaled concentrations of eight VOCs (Table 3): ammonia (18.0332 m/z), methylamine (31.0416 m/z), ethanol (47.0491 m/z), acetone (59.0491 m/z), dimethyl sulfide (DMS; 63.0263 m/z), isoprene (69.0699 m/z), pentanal (87.0804 m/z) and heptanal (115.1117 m/z).

**Table 3:** Exhaled alveolar concentrations of selected VOC in the study population.

	Ammonia [ppbV]	Methylamine [ppbV]	Ethanol [ppbV]	Acetone [ppbV]	DMS [ppbV]	Isoprene [ppbV]	Pentanal [ppbV]	Heptanal [ppbV]
Controls (n = 60)	284 ^a, b, c, d, e, f)^ (43.2–830)	10.1 ^a, b, c, d, e, f)^ (5.14–144)	82.41 ^a, b, c, d, e)^ (20.73–554.3)	232 (186–306)	10.0 ^a, b)^ (0.99–151)	49.6 ^a, b, d, e)^ (7.44–153)	5.30 ^a, b, c, d)^ (1.36–36.9)	2.78 ^a, b, c, d, e)^ (0.52–9.88)
Patients (n = 56)	556 ^a)^ (92.3–4240)	6.52 ^a)^ (1.42–29.0)	146.4 ^a)^ (15.14–1349)	226 (146–702)	14.5 (0.18–69.7)	70.5 ^a)^ (1.80–489)	9.32 ^a)^ (2.73–59.2)	5.42 ^a)^ (0.17–16.2)
KTx (n = 8)	757 ^b, h)^ (482–4240)	5.35 ^b)^ (2.01–8.32)	206.1 ^b)^ (96.78–497.9)	239 ^a)^ (217–702)	20.2 ^a)^ (12.5–69.7)	101 ^b, c)^ (43.8–489)	14.2 ^b, f)^ (8.18–30.2)	8.29 ^b, g)^ (5.82–15.0)
Conservative Treatment (n = 48)	461^c, h)^ (92.3–840)	6.61^c)^ (1.42–29.0)	137.2 ^c)^ (15.14–1349)	223 ^a)^ (146–276)	12.6 (0.18–57.7)	57.1 ^c)^ (1.80–286)	8.08 ^c, f)^ (2.73–59.2)	4.43 ^c, g)^ (0.17–16.2)
HUS (n = 9)	770 ^d, g)^ (265–840)	5.13 ^d)^ (2.58–19.7)	139.0 ^d)^ (46.42–428.7)	235 (200–276)	19.4 ^b, c)^ (7.94–57.7)	100 ^d, g)^ (24.9–286)	8.18 (2.73–15.2)	5.02 ^d, h)^ (0.66–7.87)
GD (n = 15)	690 ^e)^ (135–823)	5.94 ^e)^ (1.42–29.0)	192.5 ^e, f)^ (46.03–1349)	221 (146–248)	18.5 (3.52–37.8)	77.2 ^e, f)^ (25.5–268)	10.3 ^d, e)^ (5.87–59.2)	7.80 ^e, f, h)^ (4.13–12.3)
MF (n = 24)	425 ^f, g)^ (92.3–773)	7.26 ^f).^(2.37–24.6)	82.74 ^f)^ (15.14–1293)	222 (193–259)	11.1 ^c)^ (0.18–26.2)	46.3 ^f, g)^ (1.80–248)	7.52 ^e)^ (3.08–27.7)	2.25 ^f)^ (0.17–16.2)

Data is given as median and range. Superscripts denote significantly different concentrations of the respective analytes between identically labelled groups.

Ammonia: ^a, b, c, d, e)^ p ≤ 0.001; ^f, g)^ p < 0.01; ^h)^ p < 0.05

Methylamine: ^a, b, c, e)^ p < 0.001; ^d, f)^ p < 0.005

Ethanol: ^a, b, e)^ p ≤ 0.001; ^c)^ p < 0.005; ^d, f)^ p < 0.05

Acetone: ^a)^ p < 0.05

DMS: ^a, b, c)^ p < 0.05

Isoprene: ^a, b, c, e, f)^ p < 0.05; ^d)^ p < 0.001; ^g)^ p < 0.005

Pentanal: ^a, b, d)^ p ≤ 0.001; ^c)^ p < 0.005; ^e)^ p < 0.05; ^f)^ p < 0.01

Heptanal: ^a, b, e, f)^ p < 0.001; ^c)^ p ≤ 0.005; ^g)^ p < 0.01; ^d, h)^ p < 0.05

To account for effects of medication onto exhaled VOC profiles, we compared CKD 1 patients with and without antihypertensive drugs (S2 Table). These drugs did not have significant effects onto exhaled VOC concentrations.

**Fig 3:**
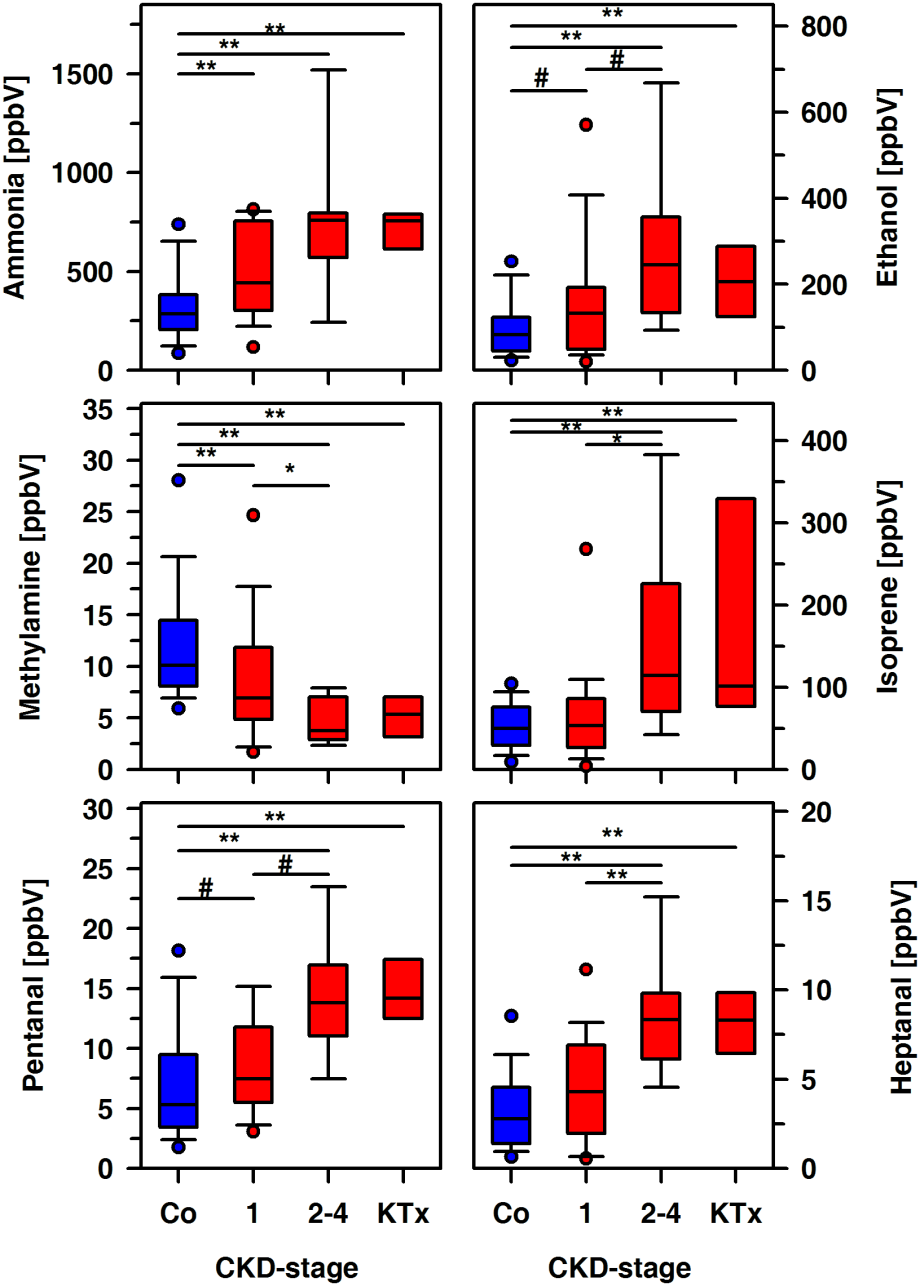
Exhaled alveolar concentrations of ammonia, ethanol, methylamine, isoprene, pentanal and heptanal in healthy controls (Co, n = 60) and CKD patients (n = 56). Box plots were used to summarize data from controls, KTx patients and those with mild (CKD stage 1) and moderate (CKD stages 2–4) disease. Significant differences between groups are indicated (**, p < 0.001; *, p < 0.01; #, p<0.05).

Patients and controls exhaled acetone and DMS in similar amounts, but remarkable differences between both groups were noted for ammonia, methylamine, ethanol, isoprene, pentanal, and heptanal (Table 3). First of all, we discriminated KTx patients and those with mild or moderate CKD, i.e. an eGFR above or below 90 ml/min/1.73m^2^. The exhaled amounts of ammonia, ethanol, methylamine, and pentanal differ significantly between controls and CKD patients with either mild or moderate disease (Fig 3). The alveolar concentrations of these compounds except of ammonia were significantly different in patients with mild and moderate CKD, respectively. Our data suggests that even a mildly impaired renal function translates into remarkable metabolic aberrations. Therefore, we considered the underlying renal diseases, i.e. HUS, GD and hereditary malformations as an alternative for categorization of the data (Table 3).

**Fig 4:**
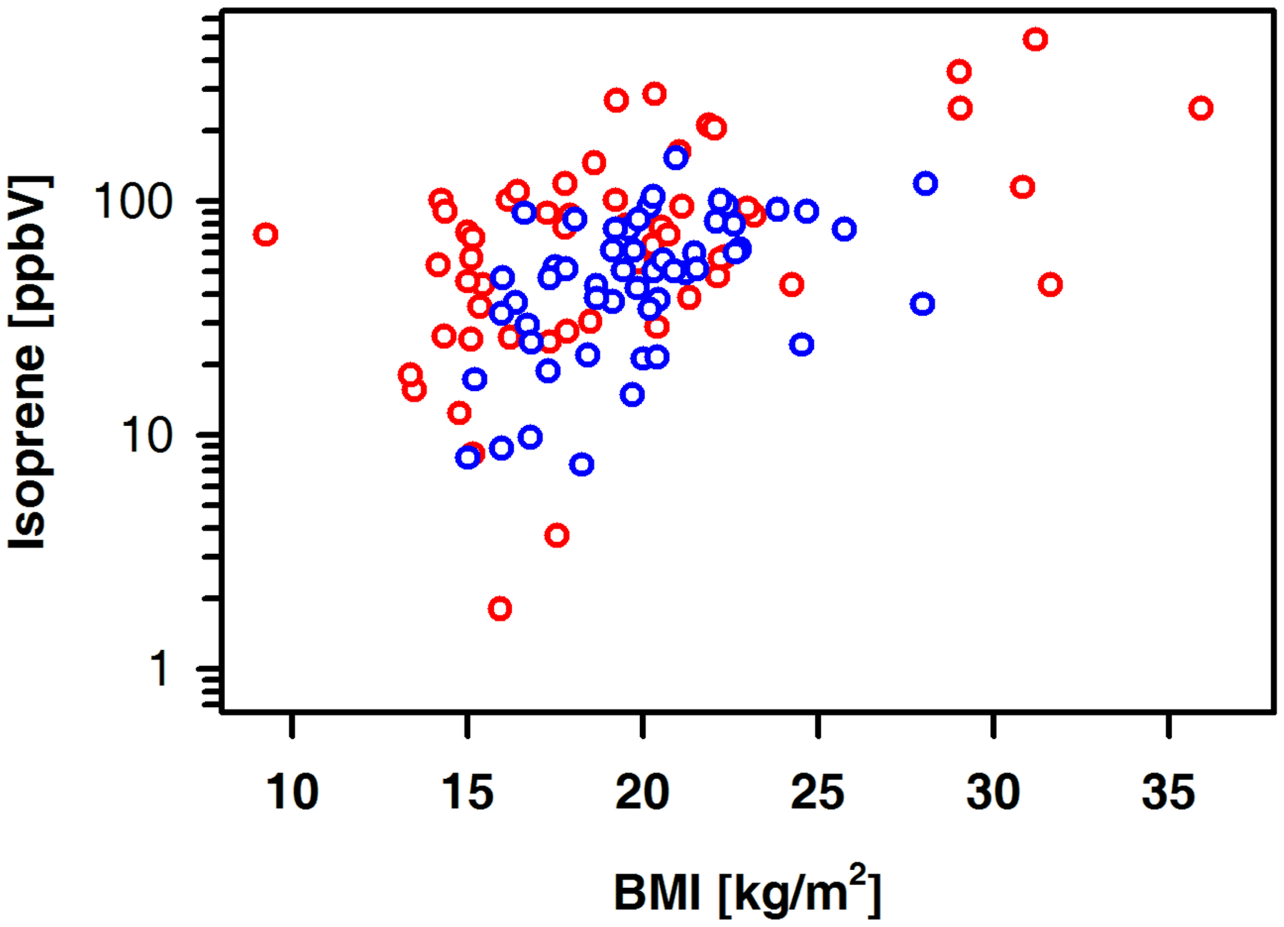
Exhaled alveolar concentrations of isoprene relative to the BMI in controls (blue circles) and patients (red circles). Controls (n = 60): r = 0.53, p < 0.001; patients (n = 56): r = 0.47, p<0.001.

**Fig 5:**
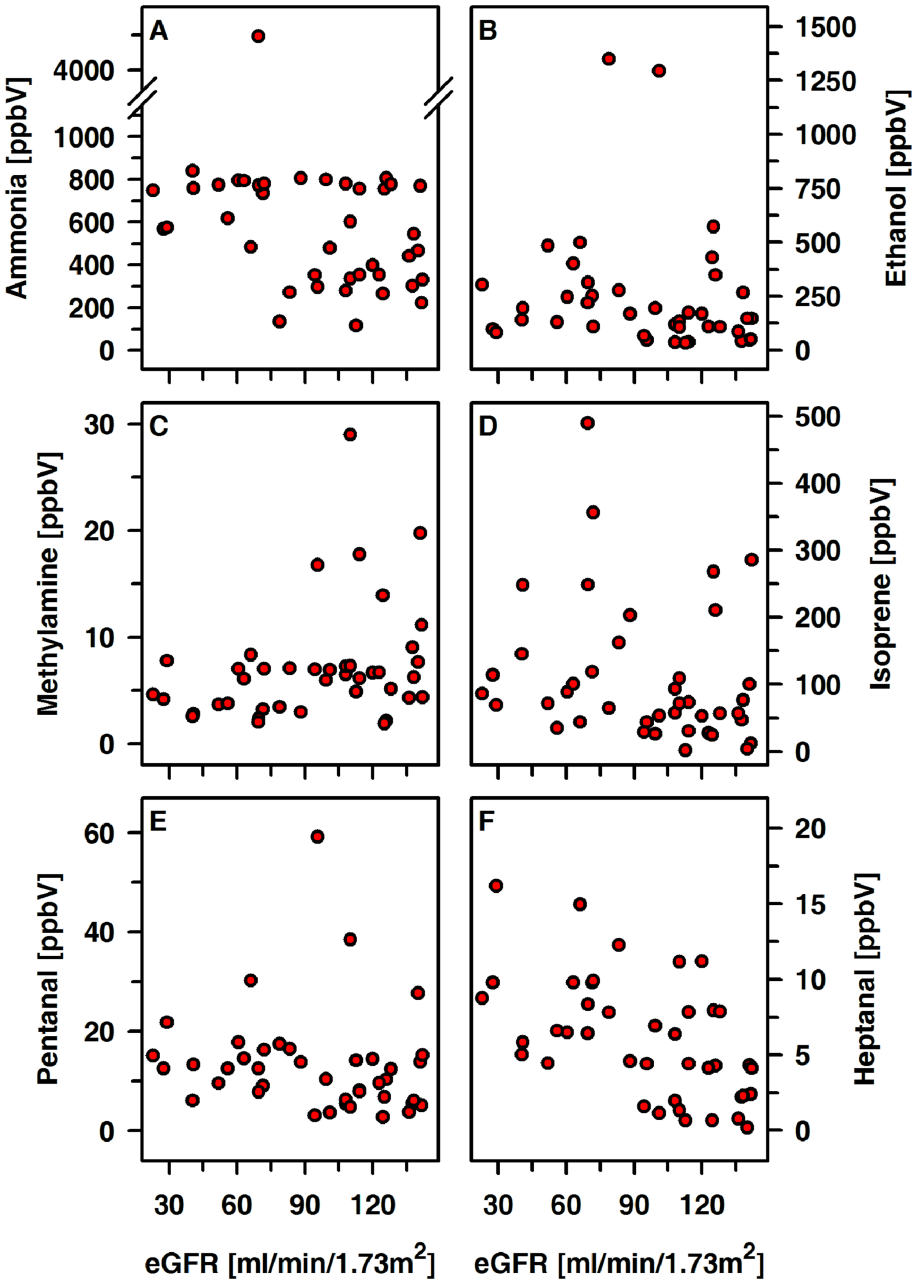
Exhaled alveolar concentrations of ammonia, ethanol, methylamine, isoprene, pentanal and heptanal relative to the eGFR in CKD patients (n = 56).

The exhaled amount of isoprene in patients and controls was positively related to age (controls: r = 0.60, p < 0.001; patients: r = 0.58, p < 0.001) and BMI (Fig 4). Such an association was not seen for ammonia, methylamine, ethanol, acetone, DMS, pentanal or heptanal. There were no correlations between ammonia, ethanol, methylamine, isoprene, pentanal, heptanal and eGFR (Fig 5).

## Discussion

In this cross-sectional study, we applied PTR-ToF-MS for real time analysis of VOCs in exhaled breath of 56 pediatric patients with mild-to-moderate CKD and 60 matched healthy controls. This technique allows for the detection of a broad panel of volatile organic compounds ("volatilome") and subsequent comparison between groups. Breath VOC data was analyzed and presented in a way similar to other “omics” technologies [21]. Out of more than 300 VOCs, the normalized concentrations of 71 substances were investigated. A heat map was used to visualize normalized data, i.e. to demonstrate per subject relative amounts of these masses without statistical evaluation regarding differences between groups. PCA was used to highlight associations within individual volatilomes and irrespective from the underlying disease. This approach led to a reduction of data and helped to focus on ammonia, methylamine, ethanol, acetone, dimethyl sulfide, isoprene, pentanal, and heptanal, which we considered as relevant even in mild to moderate CKD.

Ammonia is a key molecule in the urea cycle removing nitrogen from protein metabolism. Although our patients presented with fairly preserved renal function, exhaled ammonia concentrations were already significantly higher than in healthy controls. This indicates that already in early stages of CKD renal metabolization and clearance of nitrogen-containing substances is impaired [22,4]. Previous studies in patients with severe chronic renal failure revealed on the one hand an overall reduced renal ammonium production. On the other hand, the rate of ammonium production per unit of GFR is fourfold greater in CKD patients in comparison to healthy controls [23,24]. Correlations between concentrations of breath ammonia, blood urea nitrogen (BUN) and creatinine described in the literature [6,25] suffer from large variations which in most cases prevent a clinically relevant estimation of the above mentioned parameters. In this study, we could not detect a significant correlation between exhaled VOCs and eGFR.

Even in blood, sampling and analysis of ammonia is still challenging due to effects of potential hemolysis of red blood cells releasing additionally ammonia, metabolic changes, transport requirements and time until analysis as well as constitution of patients being anxious or doing physical exercise [26,27]. Vaziri et al. examined the effect of hemodialysis in CKD patients on blood ammonia for the first time and observed rather a rise of blood ammonia concentration probably associated with an increase of blood bicarbonate and a decrease of mean arterial pressure [28]. This is in accordance with the fact that ammonia exhalation is pH-dependent. Brock et al. reported a 33% change of [NH_4_^+^] / [NH_3_], when pH is changed by only 0.1 [29].

Ammonia can also be produced by bacteria in saliva, respiratory and gastrointestinal tract [27,30,31]. Chen et al. described that breath exhaled ammonia concentrations were significantly influenced by oral fluid urea concentrations, bacterial urease activity and mouth fluid pH [32]. Thus, the correlation between blood and breath ammonia needs further evaluation.

To our surprise and contrary to what is expected from previous studies, our CKD patients exhaled significantly less amounts of methylamine than healthy controls [33,34]. This might be due to the rather good clinical shape of our patients and in most of them accumulation of urea was not yet relevant. Given that methylamine results from desamination of adrenaline under the action of monoamino oxidase one may speculate whether healthy children perceived the breath measurement as an exciting experience with concomitant release of epinephrine. Release of epinephrine could possibly have been induced by medication via sympathetic nervous system. Since healthy controls and some of CKD stage 1 patients, who showed increased breath methylamine levels, did not take any medication, we rather associated that effect on stress or excitement.

Dimethylamine and trimethylamine have also been described as uremic toxins many times and to be exhaled in higher concentration by dialysis patients [1]. In this study, it was not possible to determine low ppbV concentrations of dimethylamine and trimethylamine by means of the PTR-ToF-MS. It remains to be elucidated whether these amines became relevant only when renal function is almost completely gone.

Isoprene is known as by-product for cholesterol biosynthesis, while body tissue represents a potential storage volume for this hydrocarbon [35,36]. Isoprene levels in healthy controls were comparable with results of Smith et al, who analyzed exhaled breath in healthy pupils [37]. In line with this study, some association between BMI and exhalation of isoprene was noted.

Remarkably highest isoprene levels were seen in KTx patients and in those with an eGFR below 90 ml/min/1.73m^2^. Categorizing the data according to the underlying disease revealed the lowest isoprene levels in patients with MF, although half of the patients with MF present with moderate CKD. Dys-/Hyperlipidemia and aberrant metabolism can be related to glomerulopathies [38] as well as to immunosuppressive therapy [39,40]. Given that patients with GD are at risk for cardiovascular disease and that patients with a history of HUS are prone to serious complications even several years after recovery, subclinical aberrations of cholesterol metabolism may already be present [41–43]. At time of the study examination the respective patients were in complete remission and statins as an indicator of a disturbed lipid metabolism are required in a minority only.

Increased concentrations of aldehydes such as pentanal and heptanal in CKD patients might mirror increased oxidative activity rather than kidney damage per se. Chronic micro inflammation and the associated oxidative stress are accused as the culprit for many if not for all comorbidities seen in CKD patients. However, verification of oxidative stress is still challenging. It remains to be elucidated whether the increased concentrations of pentanal and heptanal are due to oxidative stress and/or an impaired antioxidant response even in patients with mild-to-moderate renal failure [44,45]. It is particular interesting that exhalation of aldehydes was at maximum in kidney transplant recipients as it is tempting to speculate that in these patients oxidative stress is related to the immunosuppressive therapy [46]. As these processes are important to monitor disease progression or even to tailor therapy breath biomarkers may be useful to monitor such processes non-invasively.

Some aldehydes have also been described as contaminants from the clinical environment [5,7]. Since in our study inspiratory concentrations of pentanal and heptanal were well below alveolar levels, we could exclude this possibility.

Acetone is a marker of lipolysis and glucose metabolism. However, these pathways are not primarily affected in CKD and thus no significant differences in exhaled acetone between healthy controls and pediatric CKD patients were observed. Exhaled acetone concentrations were comparable with results of Schwarz et al [47]. Ethanol levels were increased in CKD patients in comparison to healthy controls and this might reflect the frequent exposition to a clinical environment with the accompanying high concentrations of disinfectants [48].

Thus far, sulfur-containing volatiles like dimethyl sulfide have been described in patients with end-stage renal failure [7,49]. While in KTx patients the amounts of dimethyl sulfide were significantly higher as in controls, this was not seen for patients with mild-to-moderate CKD. However, if patients were categorized according to the underlying disease, those with hereditary malformations or a history of HUS exhale significantly more DMS than healthy controls. Since DMS may also be generated by bacteria and DMS in KTx and in HUS patients were rather high, this might point to subtle changes of the gastrointestinal microbiome [50,51]. It remains to be elucidated whether this is due to immunosuppressive therapy or reminiscent to the previous bacterial infection in HUS patients.

The causes of kidney damage in our patients range from hereditary malformations to (auto-) inflammatory diseases with concomitant medications. Despite the apparently normal eGFR at time of the study investigation, the CKD 1 patients are well at risk for insidious progression of CKD and/or the development of comorbidities like hypertension, endothelial dysfunction and cardiovascular disease [41,52,53]. Although these CKD stage 1 patients have normal kidney function and suffer from a number of different pathologies our data suggests remarkable metabolic aberrations and, therefore, a potential application of breath gas analysis to detect early metabolic changes. Any chronic impairment of kidney function causes a multitude of metabolic adaptations, since the elimination of so-called uremic toxines and xenobiotics as well as electrolyte homeostasis, acid-base-control, protein turnover, and the formation/secretion of multiple hormones, e.g. calcitriol, fibroblast growth factor-23 (FGF-23) and PTH are affected. These aberrations are most severe in patients with terminal renal insufficiency and the risk of CKD associated comorbidities (e.g. cardiovascular disease, renal osteodystrophy) increases as renal function declines. Most if not all of the CKD associated complications are clinically silent for quite a long time. Nevertheless, the pathophysiological processes triggered by an impaired renal function translate into metabolic reactions which are able to maintain overall homeostasis and to compensate for mild renal dysfunction. As an example, the steep and early increase of the bone-derived phosphaturic FGF-23 keeps serum phosphate well within the normal range and at the same time triggers left ventricular hypertrophy and cardiovascular disease in CKD patients [54–56]. VOCs exhaled from patients with mildly impaired renal function, i.e. those at the very begin of the road down to terminal renal insufficiency probably mirror these processes. Thus, our findings may reflect metabolic adaptation as an early event in the time course of CKD [57–59].

Furthermore, we detected differences in VOCs between patients categorized either to the mode of therapy or to the type of renal disease leading to conservative therapy and/or follow-up in our outpatient clinic. This is particular interesting in patients with a history of shiga toxin induced HUS. These patients are at risk for long-term sequelae even after initially complete recovery from the acute phase [41].

CKD patients took a variety of medications. To assess the influence of medication on exhaled VOC profiles we compared CKD 1 patients with and without antihypertensive drugs as CKD 1 patients form a fairly homogeneous group with respect to kidney function and medication. In agreement with Blanchet et al, medication did not have significant effects onto changes in breath profiles rather than the disease itself [60].

Breath VOC profiles were different in healthy controls and the investigated CKD patients. As renal function was only mildly-to-moderately impaired in the majority of patients, other factors than renal disease are likely to contribute to the observed changes in breath VOC profiles. Life-style and/or ageing associated factors like for example obesity, dyslipidemia and type 2 diabetes mellitus are virtually not relevant in our study cohort. Thus, our findings point to early and subtle pathological processes even in early stages of CKD. Since monitoring of breath VOC profiles is completely non-invasive this approach may help to gather basic information above and beyond currently available diagnostic methods especially in pediatric CKD patients. In a perspective, monitoring of patients’ breath profiles may help to understand basic mechanisms of the disease and to decipher metabolic adaptation accompanying progression of CKD.

## Supporting information

S1 Fig Loading plot obtained in CKD patients (n = 56) and controls (n = 60). PCA loading plot (PC-1 vs. PC-2) of 71 masses (18 to 373 m/z). Masses selected for further analysis are ammonia (18.0332 m/z), methylamine (31.0416 m/z), ethanol (47.0491 m/z), acetone (59.0491 m/z), dimethyl sulfide (63.0263 m/z), isoprene (69.0699 m/z), pentanal (87.0804 m/z) and heptanal (115.1117 m/z).
(TIF)

S1 Table Detailed information on relevant medication in CKD patients with conservative therapy (cons) or after kidney transplantation (KTx). (PDF)

S2 Table Comparison of selected VOC concentrations of CKD stage 1 patients with and without antihypertensive drugs. (PDF)
