# Supplementary figures and images for: Exhaled volatile substances mirror clinical conditions in pediatric chronic kidney disease
Source: PLoS One. 2017 Jun 1;12(6):e0178745. doi: 10.1371/journal.pone.0178745 (PMC5453591; doi:10.1371/journal.pone.0178745)

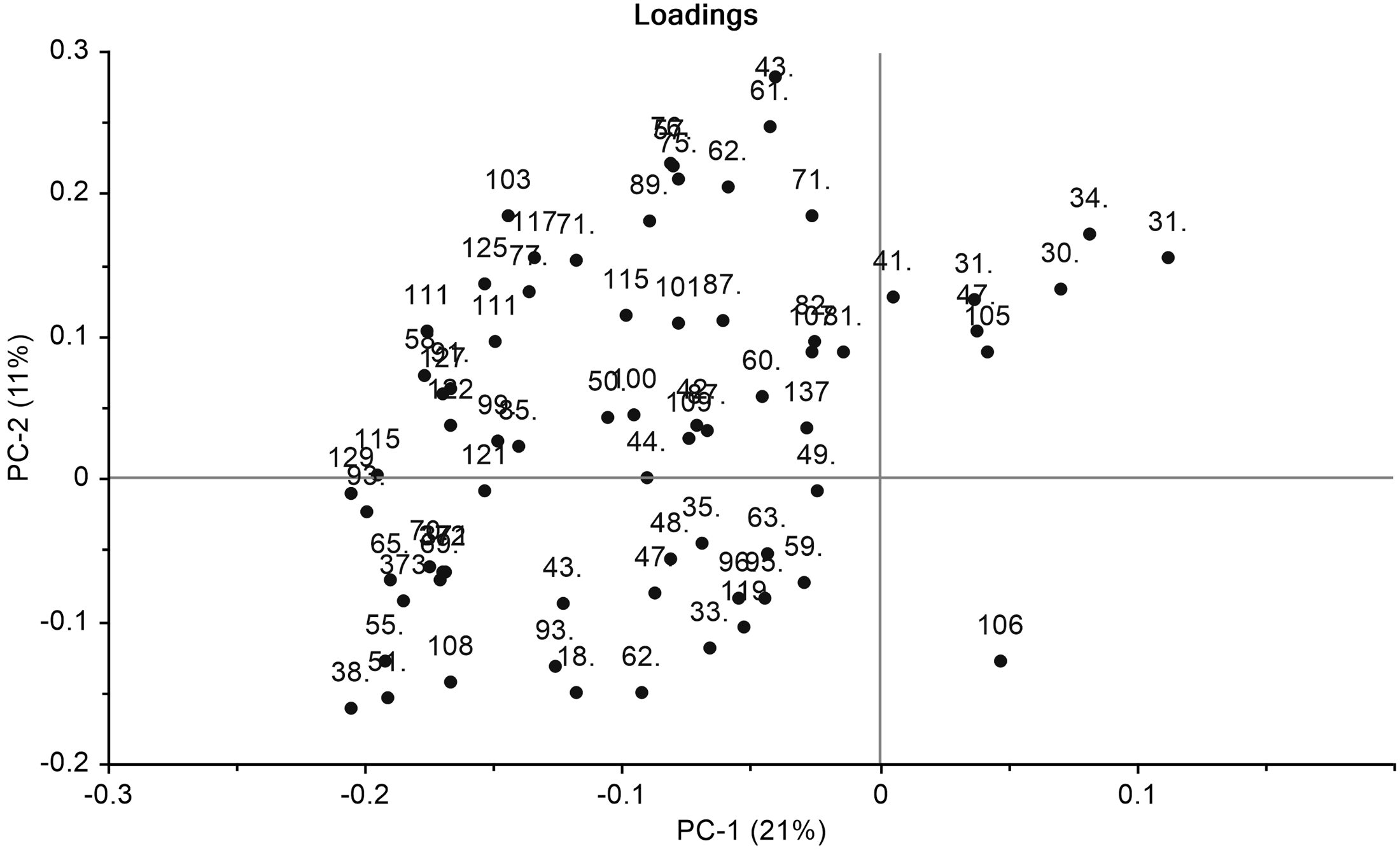

Supplement: S1 Fig — PCA loading plot (PC-1 vs. PC-2) of 71 masses (18 to 373 m/z). Masses selected for further analysis are ammonia (18.0332 m/z), methylamine (31.0416 m/z), ethanol (47.0491 m/z), acetone (59.0491 m/z), dimethyl sulfide (63.0263 m/z), isoprene (69.0699 m/z), pentanal (87.0804 m/z) and heptanal (115.1117 m/z). (TIF) [file pone.0178745.s001.tif]
