# Supplementary material for: Exhaled volatile substances mirror clinical conditions in pediatric chronic kidney disease
Source: PLoS One. 2017 Jun 1;12(6):e0178745. doi: 10.1371/journal.pone.0178745 (PMC5453591; doi:10.1371/journal.pone.0178745)
Supplement: S1 Table — (PDF) [file pone.0178745.s002.pdf]

S1 Table. Detailed information on relevant medication in CKD patients with conservative therapy (cons) or after kidney transplantation (KTx).

	**All patients**		**HUS**		**GD**		**MF**	
	cons	KTx	cons (n=9)	KTx (n=1)	cons (n=15)	KTx (n=3)	cons (n=24)	KTx (n=4)
Antihypertensive Drugs (yes/no)	26/22	8/0	6	1	11	3	9	4
ACE-Inhibitors	15	5	4	1	6	1	5	3
AT1 receptor antagonists	13	1	4		8		1	1
Beta blockers	7	5	2		1	2	4	3
Alpha blockers	2	1	1			1	1	
Vasodilators	6	4	1		2	2	3	2
Diuretics	9	6	4	1	3	1	2	4
Immunosuppressive therapy (yes/no)	12/36	8/0						
Corticosteroids	3	8		1	3	3		4
Calcineurin-Inhibitors	6			1	6	3		4
Everolimus		1						1
Mycophenolate mofetil	7	7	1	1	6	3		3
Statins	3	2	1	1	2	1		
Erythropoietin	4	3	2	1	1		1	2
Growth Hormone	1						1	
25-Hydroxyvitamin D / Calcitriol	18	7	3	1	9	2	6	4
