# Supplementary material for: Exhaled volatile substances mirror clinical conditions in pediatric chronic kidney disease
Source: PLoS One. 2017 Jun 1;12(6):e0178745. doi: 10.1371/journal.pone.0178745 (PMC5453591; doi:10.1371/journal.pone.0178745)
Supplement: S2 Table — (PDF) [file pone.0178745.s003.pdf]

S2 Table. Comparison of selected VOC concentrations of CKD stage 1 patients with and without antihypertensive drugs.

	**Antihypertensive Drugs in CKD stage 1 patients**	
	No (n = 22)	Yes (n = 17)
Age	9.00 (4.0 - 17.0)	12.0 (5.00 - 18.0)
absolute BMI [kg/m^2]	17.7 (13.4 - 22.3)	19.5 (14.2 - 24.3)
standardized BMI [SDS]	-0.09 (-1.90 - 1.81)	0.12 (-2.20 - 1.92)
Duration of disease [y]	5.92 (0.58 - 16.7)	3.92 (0.17 - 12.3)
Urea [mmol/l]	3.44* (1.79 - 7.38)	5.88* (2.64 -9.37)
eGFR [ml/min/1.73m^2]	141 (99 - 200)	126 (94 - 182)
Conservative treatment / KTx	22	16 / 1
HUS / GD / MF / KTx	3 / 4 / 15	4 / 9 / 4
Immunosuppressive therapy (y/n)	3 / 19	8 / 9
Statins (y/n)	0 / 22	1 / 16
Ammonia [ppbV]	404 (92.3 - 799)	457 (265 - 823)
Methylamine [ppbV]	7.26 (1.91 - 24.6)	6.65 (1.42 - 29.0)
Ethanol [ppbV]	107.1 (15.14 - 1293)	132.0 (46.03 - 428.7)
Acetone [ppbV]	225 (202 - 259)	228 (146 - 702)
DMS [ppbV]	11.8 (3.52 - 57.7)	15.1 (4.77 - 69.7)
Isoprene [ppbV]	44.5 (1.80 - 268)	73.1 (8.30 - 286)
Pentanal [ppbV]	7.30 (3.29 - 27.7)	8.18 (2.73 - 59.2)
Heptanal [ppbV]	2.29 (0.17 - 9.78)	4.40 (0.66 - 11.2)
